# Supplementary material for: Effect of Dietary Soy Protein Source on Effluent Water Quality and Growth Performance of Rainbow Trout Reared in a Serial Reuse Water System
Source: Animals (Basel). 2023 Oct 3;13(19):3090. doi: 10.3390/ani13193090 (PMC10571907; doi:10.3390/ani13193090)
Supplement: Supplementary file 1 [file animals-13-03090-s001.zip › animals-2590750-supplementary.pdf]

Supplementary Figures S1–S14

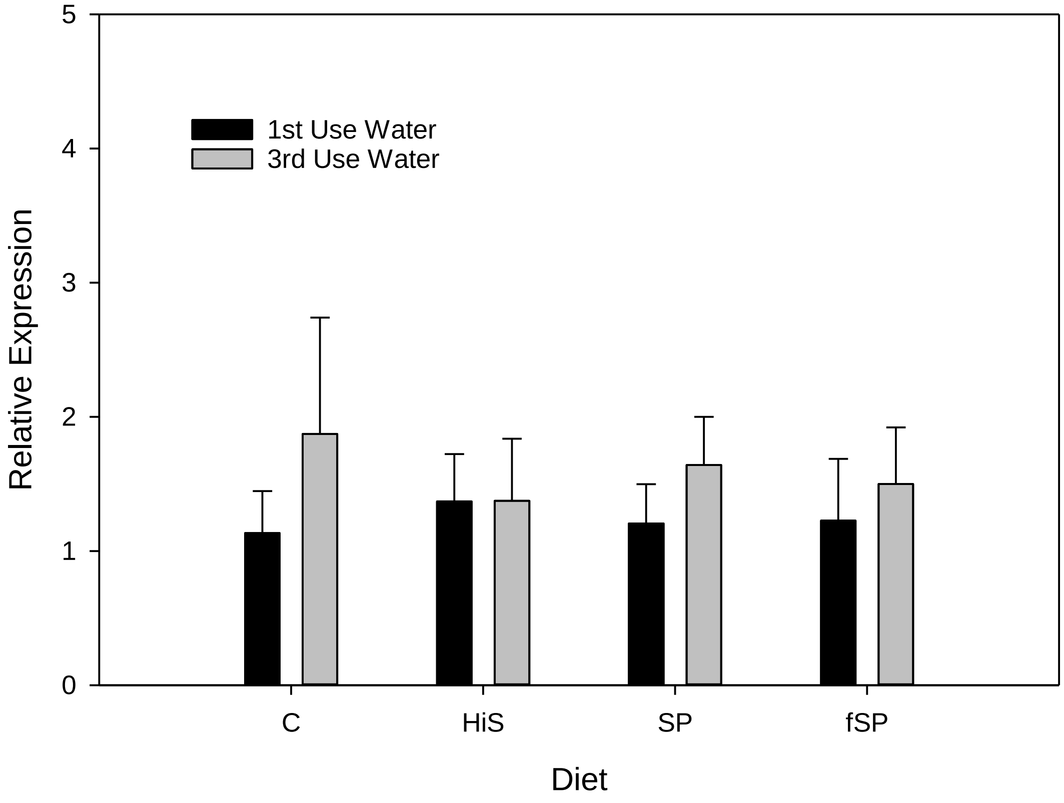

Figure S1. Relative expression (versus the reference gene, β-actin) of IRF-1 in gill of rainbow trout fed different experimental feeds and reared in 1st use or 3rd use water. C = fishmeal control; fSP = fermented soy protein concentrate; HiS = high soybean meal; SP = soy protein concentrate.

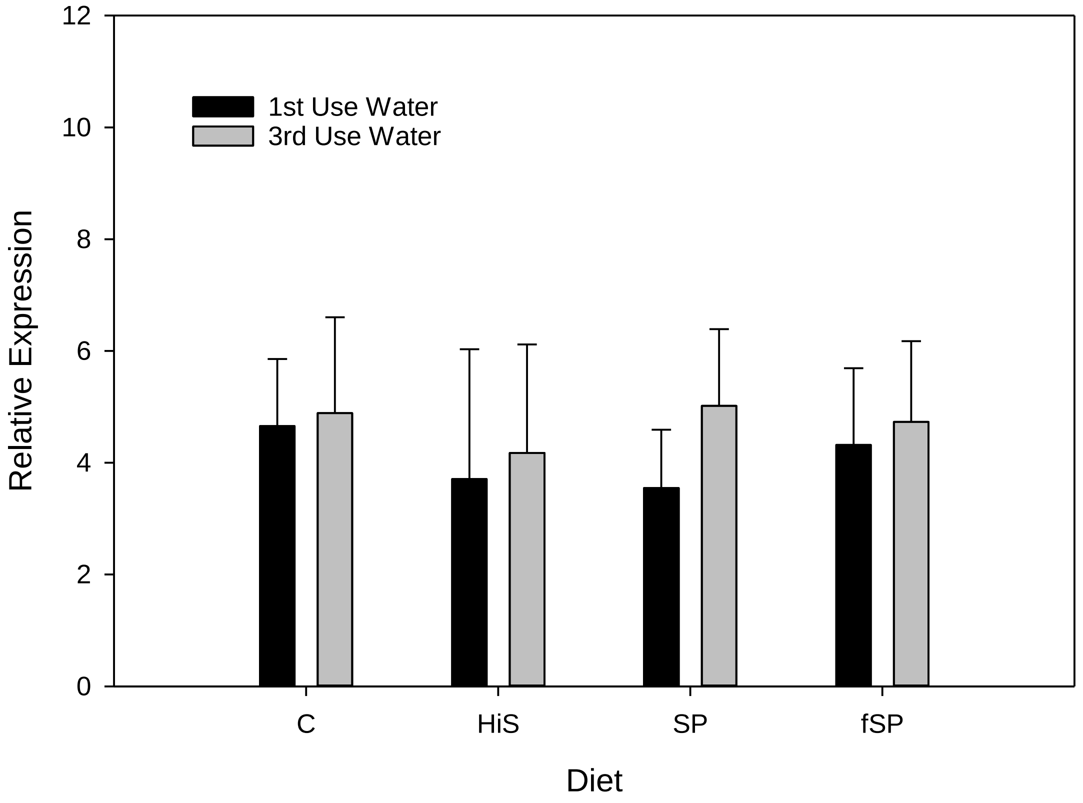

Figure S2. Relative expression (versus the reference gene, β-actin) of FK506BP2 in gill of rainbow trout fed different experimental feeds and reared in 1st use or 3rd use water. C = fishmeal control; fSP = fermented soy protein concentrate; HiS = high soybean meal; SP = soy protein concentrate.

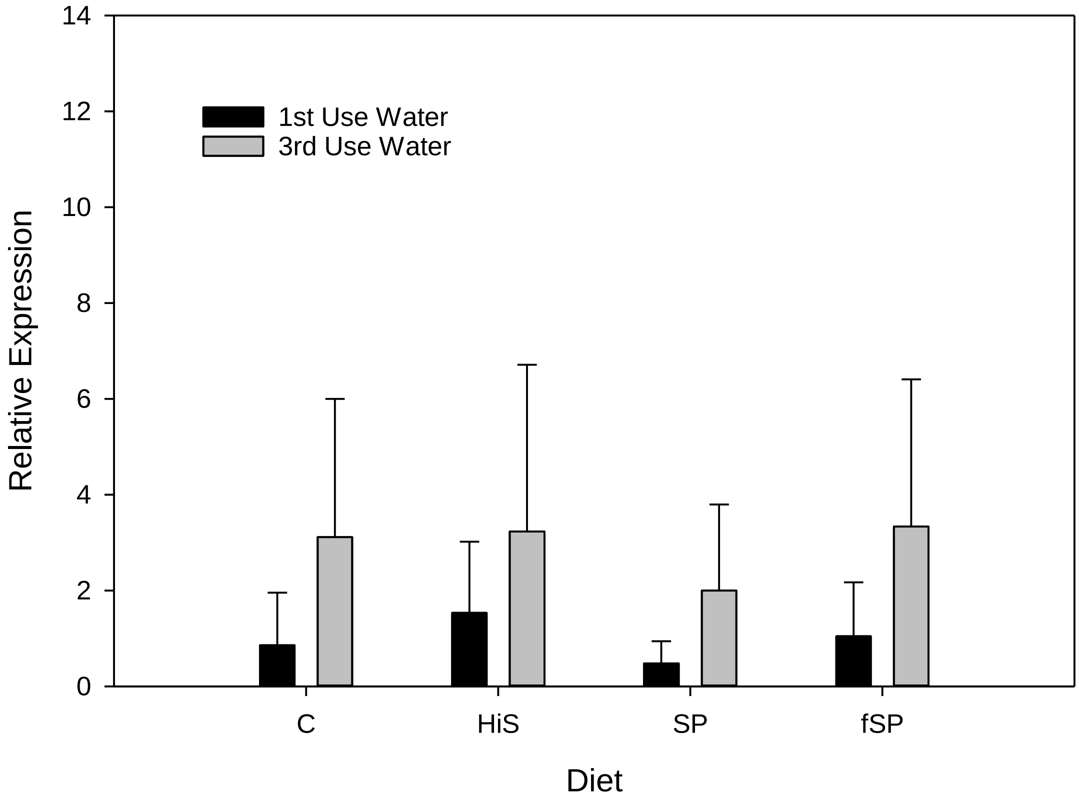

Figure S3. Relative expression (versus the reference gene, β-actin) of DIO2 in gill of rainbow trout fed different experimental feeds and reared in 1st use or 3rd use water. C = fishmeal control; fSP = fermented soy protein concentrate; HiS = high soybean meal; SP = soy protein concentrate.

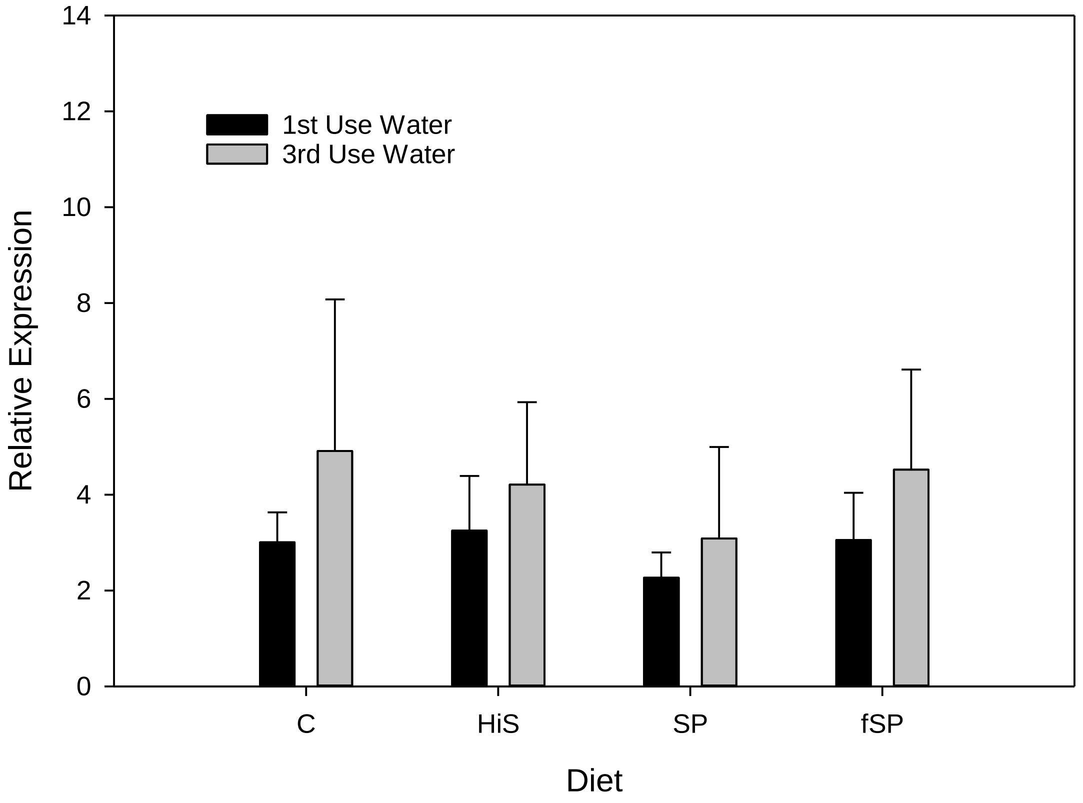

Figure S4. Relative expression (versus the reference gene, β-actin) of REGPS in gill of rainbow trout fed different experimental feeds and reared in 1st use or 3rd use water. C = fishmeal control; fSP = fermented soy protein concentrate; HiS = high soybean meal; SP = soy protein concentrate.

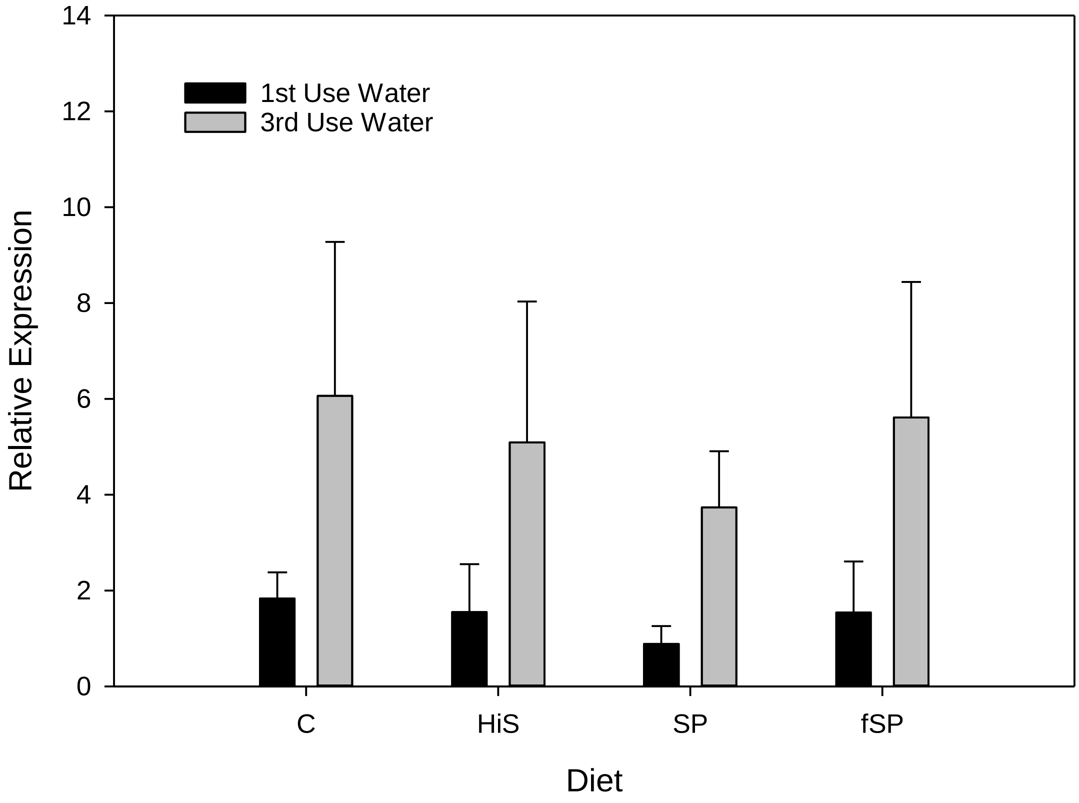

Figure S5. Relative expression (versus the reference gene, β-actin) of CYP1a in gill of rainbow trout fed different experimental feeds and reared in 1st use or 3rd use water. C = fishmeal control; fSP = fermented soy protein concentrate; HiS = high soybean meal; SP = soy protein concentrate.

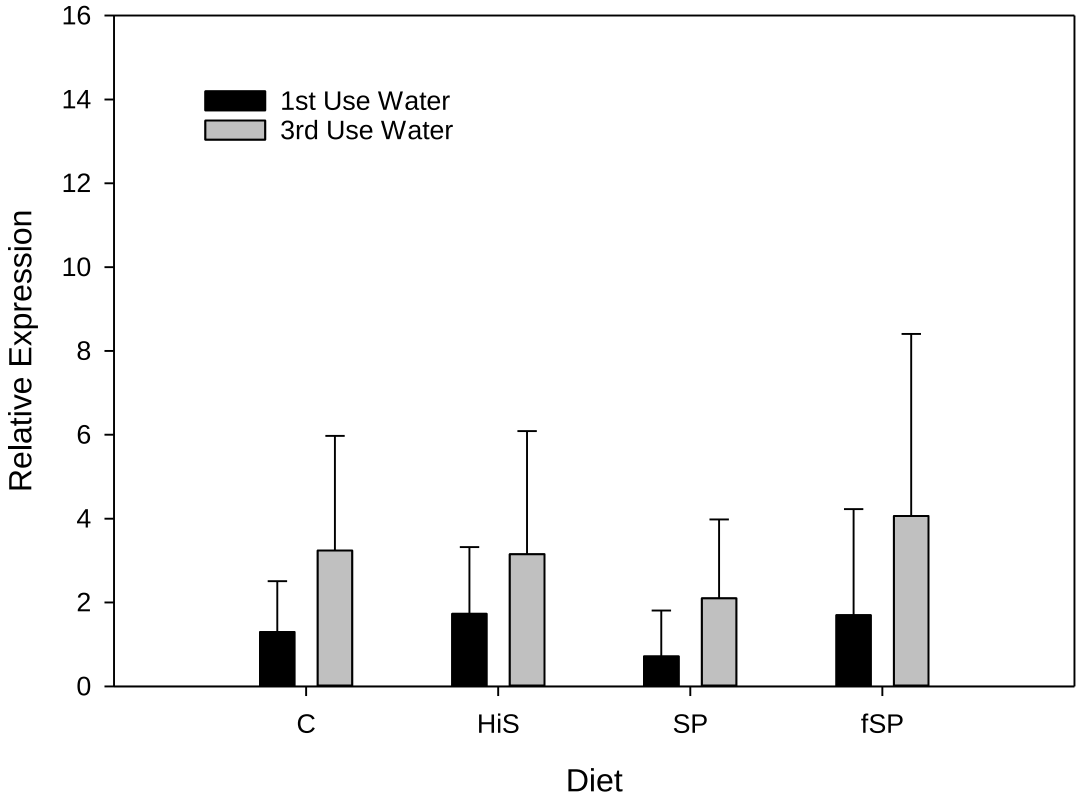

Figure S6. Relative expression (versus the reference gene, β-actin) of G6PH in gill of rainbow trout fed different experimental feeds and reared in 1st use or 3rd use water. C = fishmeal control; fSP = fermented soy protein concentrate; HiS = high soybean meal; SP = soy protein concentrate.

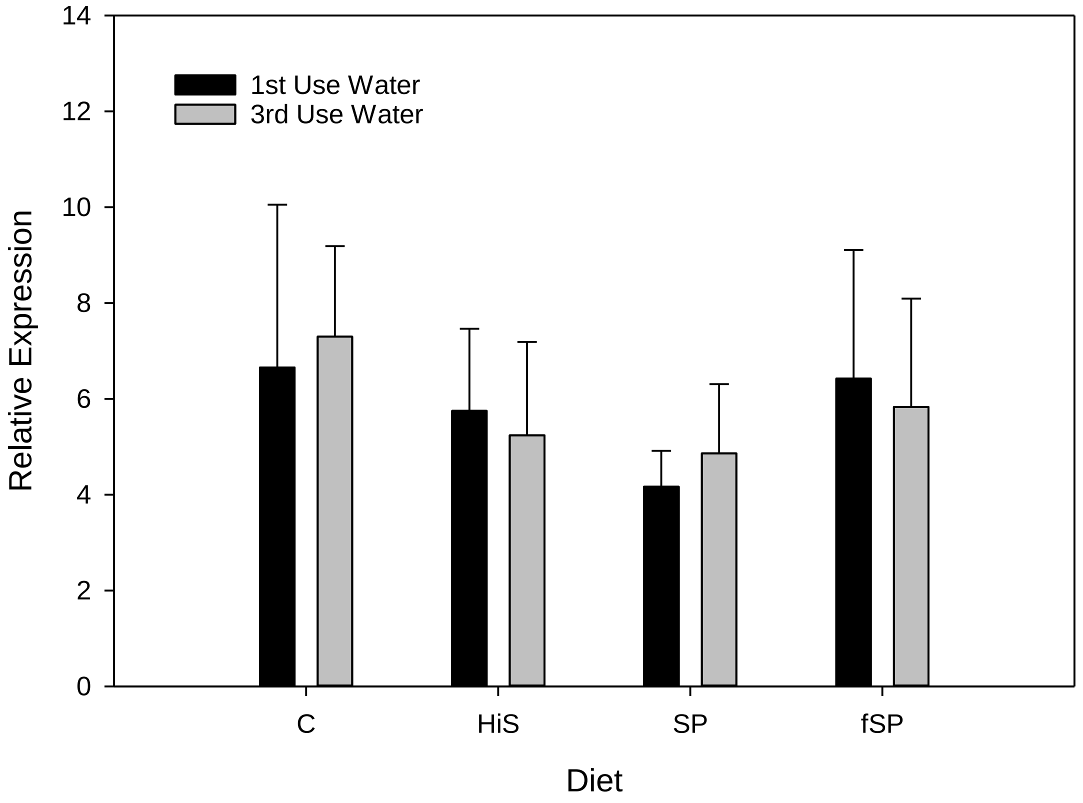

Figure S7. Relative expression (versus the reference gene, β-actin) of GADD45A in gill of rainbow trout fed different experimental feeds and reared in 1st use or 3rd use water. C = fishmeal control; fSP = fermented soy protein concentrate; HiS = high soybean meal; SP = soy protein concentrate.

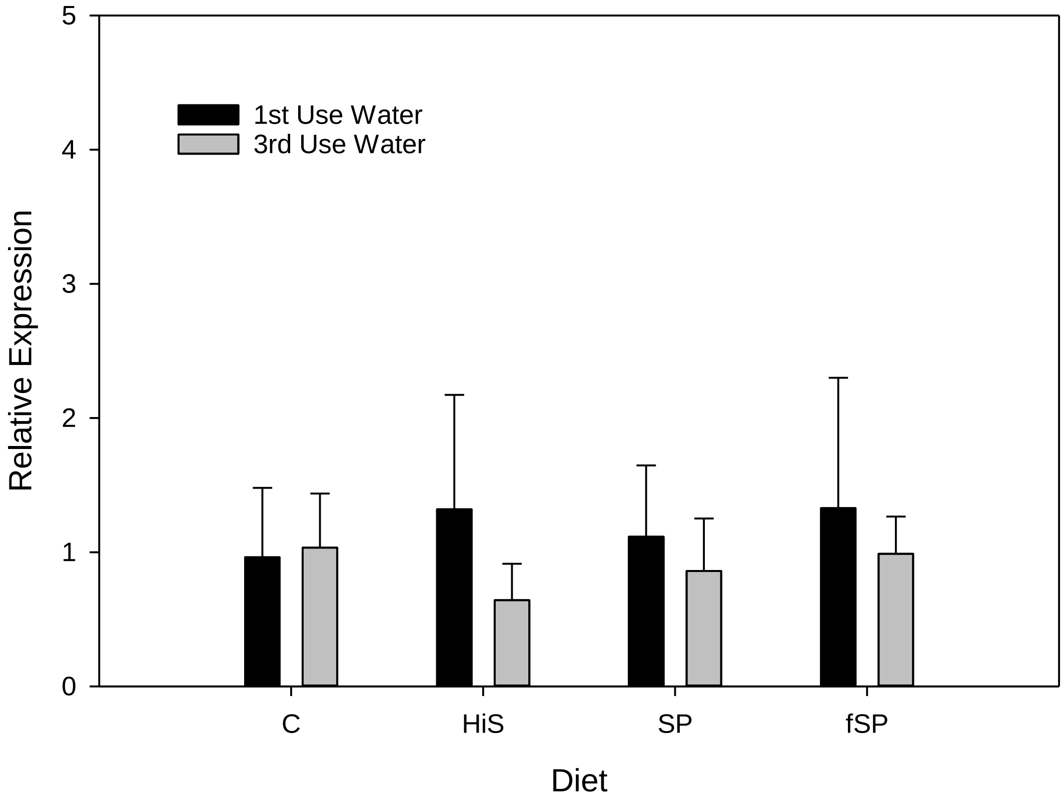

Figure S8. Relative expression (versus the reference gene, β-actin) of IRF-1 in liver of rainbow trout fed different experimental feeds and reared in 1st use or 3rd use water. C = fishmeal control; fSP = fermented soy protein concentrate; HiS = high soybean meal; SP = soy protein concentrate.

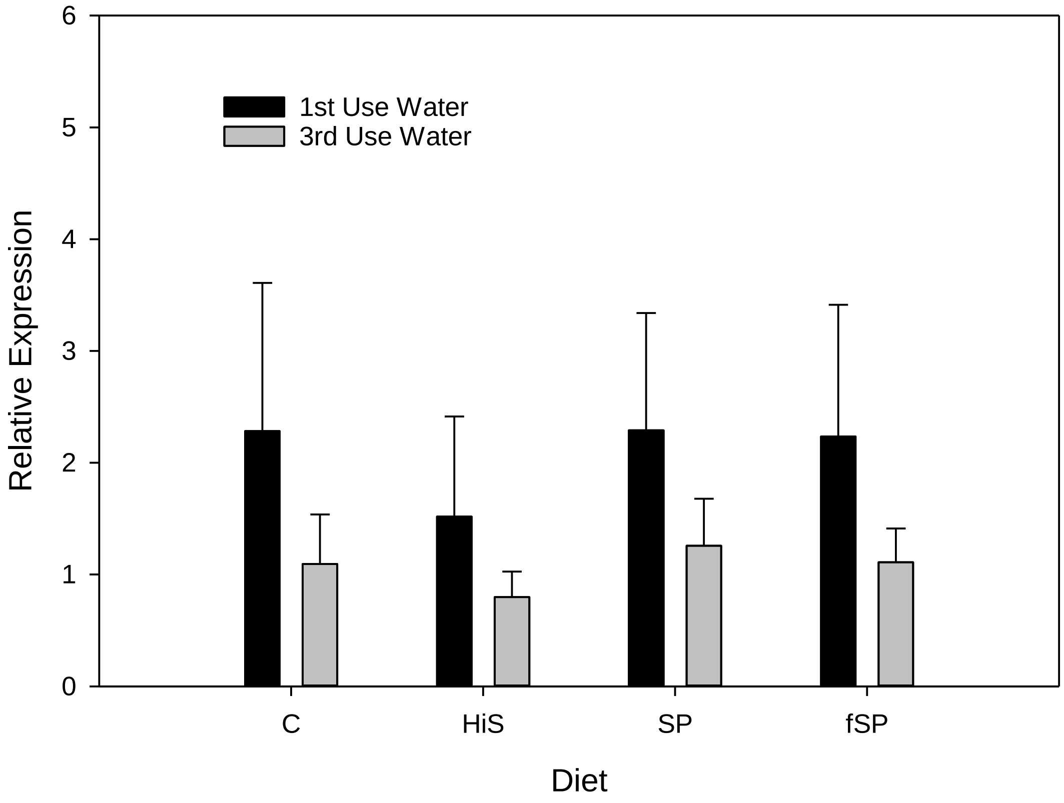

Figure S9. Relative expression (versus the reference gene, β-actin) of FK506BP2 in liver of rainbow trout fed different experimental feeds and reared in 1st use or 3rd use water. C = fishmeal control; fSP = fermented soy protein concentrate; HiS = high soybean meal; SP = soy protein concentrate.

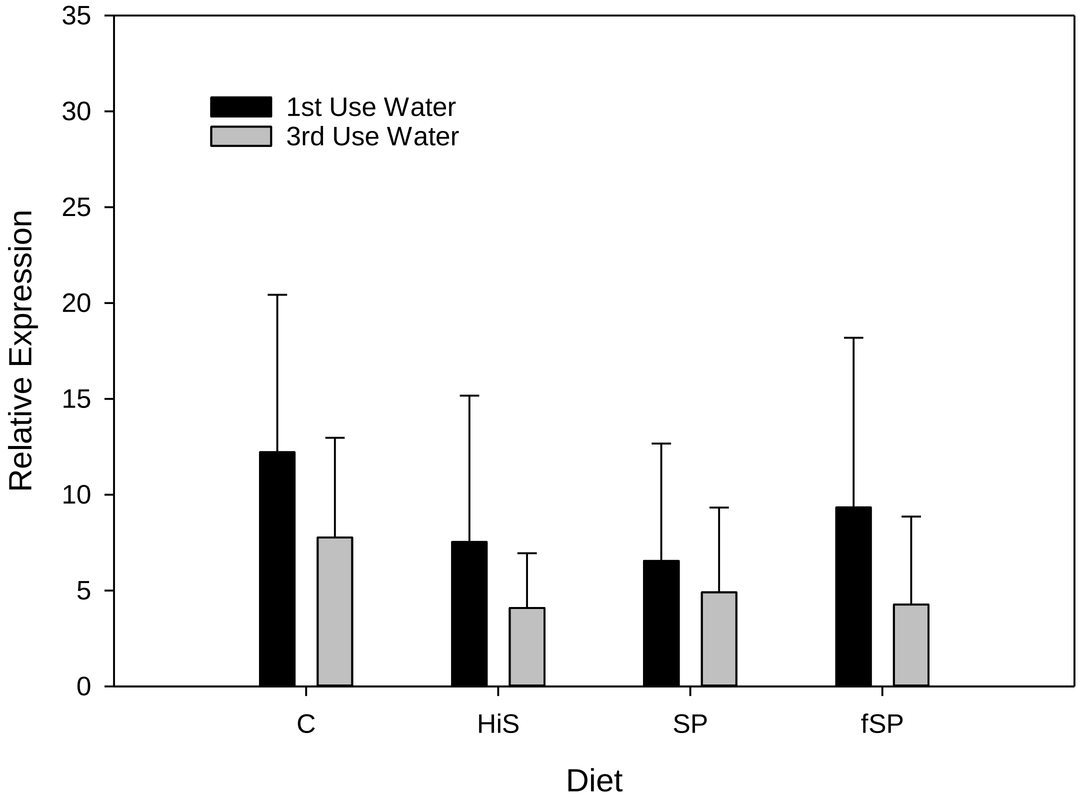

Figure S10. Relative expression (versus the reference gene, β-actin) of DIO2 in liver of rainbow trout fed different experimental feeds and reared in 1st use or 3rd use water. C = fishmeal control; fSP = fermented soy protein concentrate; HiS = high soybean meal; SP = soy protein concentrate.

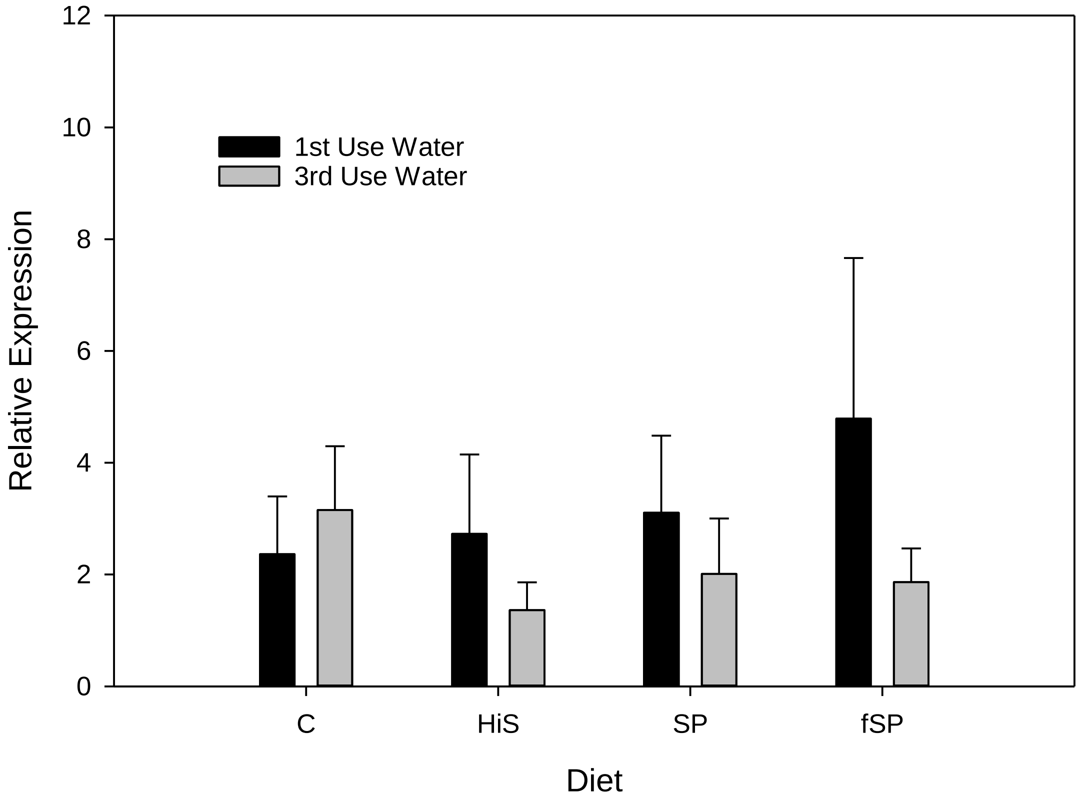

Figure S11. Relative expression (versus the reference gene, β-actin) of REGPS in liver of rainbow trout fed different experimental feeds and reared in 1st use or 3rd use water. C = fishmeal control; fSP = fermented soy protein concentrate; HiS = high soybean meal; SP = soy protein concentrate.

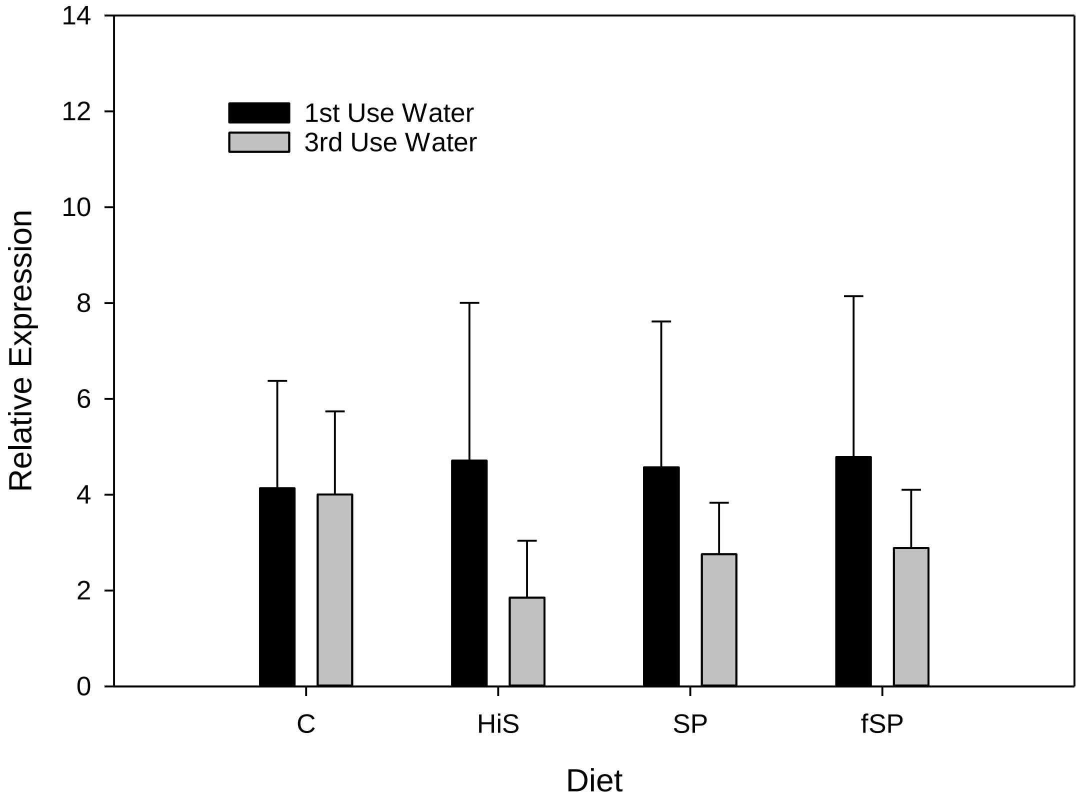

Figure S12. Relative expression (versus the reference gene, β-actin) of CYP1a in liver of rainbow trout fed different experimental feeds and reared in 1st use or 3rd use water. C = fishmeal control; fSP = fermented soy protein concentrate; HiS = high soybean meal; SP = soy protein concentrate.

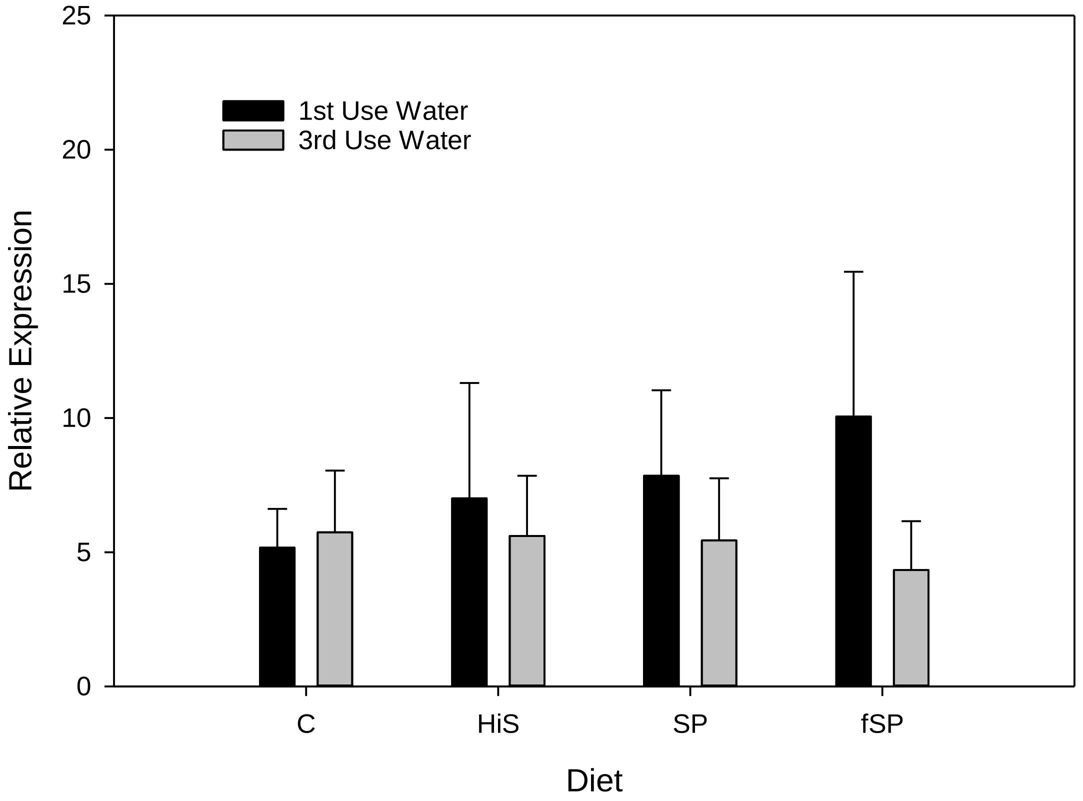

Figure S13. Relative expression (versus the reference gene, β-actin) of G6PH in liver of rainbow trout fed different experimental feeds and reared in 1st use or 3rd use water. C = fishmeal control; fSP = fermented soy protein concentrate; HiS = high soybean meal; SP = soy protein concentrate.

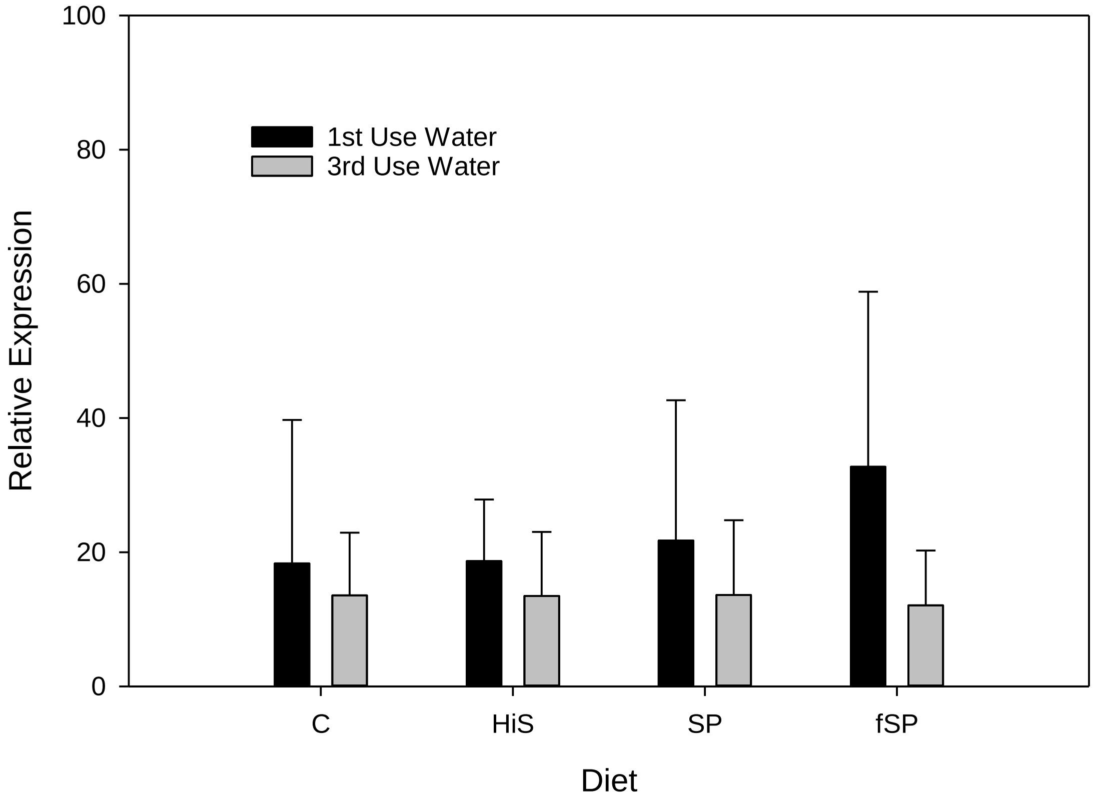

Figure S14. Relative expression (versus the reference gene, β-actin) of GADD45A in liver of rainbow trout fed different experimental feeds and reared in 1st use or 3rd use water. C = fishmeal control; fSP = fermented soy protein concentrate; HiS = high soybean meal; SP = soy protein concentrate.
